# The Biomechanics of Nickel Titanium Instrument Fracture in Root Canal Therapy: A Narrative Review

**DOI:** 10.3390/ma17246147

**Published:** 2024-12-16

**Authors:** Kushagra Ohri, Philip Yuan-Ho Chien, Ove Andreas Peters

**Affiliations:** School of Dentistry, The University of Queensland, Herston, QLD 4006, Australia; k.ohri@uq.edu.au (K.O.); philip.chien@uq.edu.au (P.Y.-H.C.)

**Keywords:** nickel titanium, austenite, martensite, fatigue, endodontics, root canal therapy, root canal preparation, torsional force

## Abstract

The use of motorized nickel titanium instruments is an essential component in contemporaneous clinical endodontics. The mechanical properties of nickel titanium are optimal for the cleaning and shaping of root canal systems. However, instrumentation carries risks, particularly instrument fracture, which may become an obstacle to achieving adequate disinfection of the canal system. Although the biomechanics of instrument fracture have been extensively investigated previously, certain facets remain unexplored, specifically torsional fatigue. This review presents a comprehensive overview of the modern understanding of the biomechanics involved in nickel titanium instrument fracture. Importantly, while research has tended to describe fatigue fracture and torsional failure as distinct and separate entities, clinical conditions are likely a combination of the two. Moreover, intracanal instrument fractures may also occur following a different mechanism, torsional fatigue. This should be taken into consideration for further research and clinical guidance.

## 1. General Introduction

Apical periodontitis is driven by the presence of microorganisms in the root canal system [1]. Management of endodontic disease consequently has focused on the removal of these microorganisms, essentially by chemical and physical disinfection of the root canal space. Contemporary root canal treatment utilizes mechanical action, in rotational or translational movements, to shape the root canal system, to allow for chemical disinfection. With the introduction and development of instruments made of nickel titanium (NiTi) alloy in the last two decades, there has been a shift toward the use of motorized rotary instruments instead of manual instruments, which has been comprehensively reviewed [2].

Current strategies aim to achieve the resolution of apical periodontitis while conserving the radicular dentine as much as possible [3]. While this approach suggests less of a reliance on unimpeded, straight-line access, the risk of instrument fracture due torsional fatigue may in fact increase. With an aging population increasingly presenting for root canal treatment, it is anticipated that the odds of presentation of teeth with narrow and calcified canals in endodontic clinical practice might also increase. Such conditions provide a challenge, since instrument fracture may occur via both cycle fatigue and torsional loading [4]. The current best evidence suggests that instruments fabricated from post-manufacture, heat-treated nickel titanium perform better compared to earlier designs in terms of flexural fatigue. However, these more martensitic instruments do not perform as well in terms of torsional resistance, mainly due to their inability to provide the same range of stress-induced superelastic transformation than their austenitic counterparts [5].

## 2. Instrumentation in Endodontics

### 2.1. The Role of Instrumentation in Endodontics

The root canal space and its complexities harbor microorganisms; the treatment of endodontic disease consequently demands their removal by thorough chemical and physical disinfection of the root canal space.

Debridement, in other words, physical disinfection, requires the preparation of the canal system using mechanical instruments such as endodontic files. Canal shaping is also essential in contemporary therapy to facilitate root canal obturation. Importantly, mechanical instrumentation cannot remove all the infected tissue from the root canal space, and instruments have been reported to contact only 65% of the root canal dentine wall [6,7]. However, mechanical shaping alone reduces bacterial load and promotes chemical disinfection, both of which are conducive to periapical healing [6]. However, the use of instruments like rotary endodontic files carries some risk.

### 2.2. The Challenges of Instrumentation in Endodontics

The root canal system often has narrow dimensions that are typically less than 1mm in cross section. As early as the 1920s, Hess showed the complexities of the root canal system, prior to any form of advanced imaging. As such, the use of rotating instruments driven by a motor carries risks: primarily, that of instrument fracture. Despite the appropriate use within the specifications of the manufacturer, instruments can fracture without the file showing any obvious signs of damage, through a process known as fatigue. Fractured fragments of instruments have the potential to hinder disinfection of the root canal system in its entirety, and their retrieval is often associated with the loss of substantial and vital amounts of tooth structure.

Since most root canals are curved in at least one plane, one major reason for the fracture of these instruments is flexural fatigue. Extensive research has led to advancements in NiTi material, which is more resistant to flexural fatigue. As a result, there are numerous NiTi instruments available which vary significantly in their mechanical properties. The impact of this difference on flexural fatigue is now well established [8]. However, torsional fatigue has been mainly unexplored. Torsional fatigue is experienced by a rotating instrument as it progresses apically when mechanically shaping a narrow or calcified canal. Because the instrument is driven by a motor attached to its shank, the instrument repeatedly twists at the cutting front before it overcomes the resistance of root canal space and cuts through radicular dentin. This repetitive twisting subjects instruments to torsional fatigue.

Over the years, the superiority of rotary endodontic NiTi over stainless steel (SS) files has become apparent because of their ease of use and ability to prepare root canal systems in a more time-efficient manner. Studies have shown that less experienced clinicians and undergraduate dental students can shape root canals in less time and with a lower frequency of iatrogenic errors with the use of NiTi files, compared to manual stainless-steel files. Despite these benefits, the use of NiTi files is associated with a higher risk of fracture, irrespective of the level of experience of the clinician.

On the other hand, NiTi files can enlarge even curved root canals to sizes that are not routinely attainable with stainless steel files, particularly in the apical third of the root canal system. Although larger shapes often lead to better irrigation of the canal system, the evidence for improved disinfection with such larger spaces is lacking [7].

The incidence of file fracture reported in the literature is relatively low. One study that analyzed clinical records and radiographs reported that 0.74% instruments fractured during primary treatment and 2.96% during retreatment [9]. Similarly, it was reported that the overall incidence of instrument fracture at an endodontic resident clinic was 0.39% [10]. However, these figures could underestimate the problem, as the majority of files that fracture do break at 1.5mm from tip, and are small enough for the event to go unnoticed by the clinician [11]. On the other hand, the incidence of instrument fracture, when assessing all the instruments used in a clinical setting under a microscope, has been reported to be between 5% and 14%; this includes the risk of fracture due to torsional resistance failure, as the use of rotary files has been associated with higher torque generation [12,13,14].

Retained file fragments may impact the goal of endodontic treatment of cleaning and shaping, and may impact the adequacy of disinfection to eliminate microorganisms [15]. Fractured instruments within the root canal that cannot be bypassed limit the access to the space past the instrument, and may negatively impact the ability to disinfect the canal space in its entirety. This is especially true for teeth with radiographic signs of periapical pathosis, for which success rate has been shown to be lower by up to 14% in teeth with retained instrument fragments [16]. Moreover, attempts to remove fractured instruments often require excessive removal of root dentin, which also increases the risk of root perforation, and has been shown to compromise the integrity of the tooth, predisposing it to fracture [17].

## 3. Fracture Mechanics of Metals

To better appreciate the file fracture mechanism described in Section 5, some basic mechanical properties of the metal require in-depth understanding. The definitions are outlined in Table 1.

### Fatigue in Metals

The term “fatigue” as related to metal was first used by Braithwaite in 1854, and the first fatigue experiments were performed by Wöhler, published in German in 1858 [21]. Early data showed that complete fracture and cracks can develop by repetitive stress far below the ultimate strength and even the yield-point of the metal [21]. Experimental evidence from Ewing and Humfrey suggested that damage from fatigue is a result of fatigue crack nucleation, which happens as microcracks within slip bands [22]. These thin bands are formed because of the localization of the cyclic strain into thin bands that run parallel to the low-index crystallographic planes, and are called persistent slip bands [23].

It is now understood that fatigue failure happens in four phases, which are as follows [24]:

- Crack nucleation
- Structurally dependent crack propagation (often called the “short crack” or “small crack” phase)
- Crack propagation that is characterizable by either linear elastic fracture mechanics, elastic–plastic fracture mechanics, or fully plastic fracture mechanics
- Final instability

Crack nucleation and the propagation rate determine the overall fatigue life of the material. However, where the stress amplitude places the cycling strain within the elastic strain limits below the yield point, the crack initiation phase dominates the fatigue life of the material [24].

Crack propagation or growth has been considered as a two-stage process [21], wherein a crack begins as a microcrack (similar to crack nucleation), and it extends within the slip bands on a plane of high shear. A Stage 1 microcrack becomes a Stage 2 macrocrack when it reaches the critical length, changes direction, and propagates normal to the principal stress [21,25]. The so-called critical length is not only dependent on the length of the crack, but also on the microstructural features of the material, and, more importantly, on the stress-intensifying factors at and around the developing microcrack [26]. All of this occurs continuously with cyclic loading and as microcracks grow.

At the atomic level, since almost all metals exist in crystalline form, slip bands form because of the movement of dislocations in the crystals at below the ultimate stress [27]. Taylor proposed that the energy required for the breaking and reforming of atomic bonds (one or few at a time) for a row of atoms in response to shear stress is much lower than that required for the breaking of all the bonds on an entire plane of atoms at once, resulting in some plastic deformation at the microscopic level, caused by dislocations, which become carriers for plastic deformation [27]. Heat treatment, alloy content, and cold working can change the number and arrangement of the dislocation population and how they move and interact to create useful properties [28,29].

**Figure 1:**
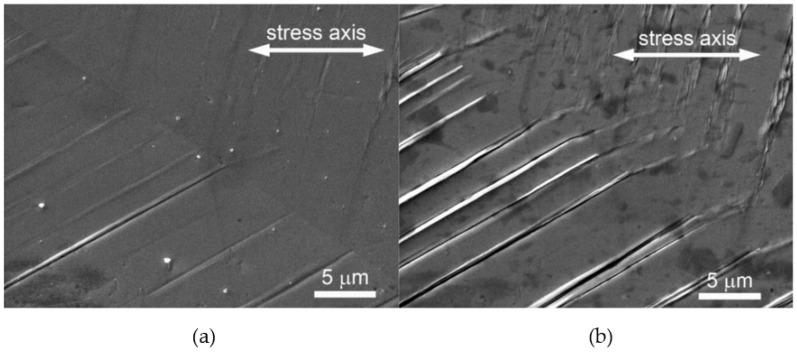
Evolution of PSMs in a grain of polycrystalline copper cycled with low strain amplitude (ε_a_ = 1 Å~10^−3^), N*_f_* = 10^5^, (**a**) N = 5% N*_f_*, (**b**) N = 50% N*_f_*. Reproduced from Polak 2023 under the Creative Commons Attribution (CC BY) license [35].

Localized plastic straining is observed as extrusions and intrusions on the surface of fatigued metals [30]. These extrusion and intrusions are visible in SEM images (Figure 1) as persistent slip markings (PSMs), formed from persistent slip bands because of the surface relief where the slip bands egress on the surface [31]. These slip bands are persistent in the sense that in an experimental set-up, they reappear even after the interruption of fatigue cycling and repolishing, and after new cycling [32]. During cyclic straining, plastic strain concentrates in the slip bands, and the plastic strain amplitude can increase more significantly in the slip bands than the average applied strain amplitude [23,33].

At the tip of the intrusions, and in the areas between regularly spaced intrusions, local slip intensification occurs. Moreover, the restraint on the slip is much further toward the surface than within the material, so together with enhanced slip intensification, it leads to the initiation of microcracks from those on the surface of metal [34]. With the effect of subsequent slip intensifications at the crack tip, the crack formation accelerates. This suggests that the surface relief enhances the irreversible slip and nucleation of shallow cracks [32].

There is some disagreement regarding the mechanism leading to the formation of Stage 1 cracks. The Essmann-Gösele-Mughrabi (EGM) model ascribes a passive role to intrusions formed on the surface, contrary to Polak’s model [29]. The EGM model suggests that intrusions do not develop from a similar mechanism to extrusion, and are less dominant. According to this model, vacancies are generated within the walls of the dislocation slip bands, and this expands the volume of the slip bands. This volume expansion results in slip band lamella elongation and extrusion of the material at the surface. Subsequently, this causes the initiation of Stage 1 cracks, in the form of intrusions, on both sides of the microscopically extruded material, due its notch effect at the slip band–matrix interface [29,36].

Conversely, Polak’s model suggests that there is redistribution of atoms from the metal matrix surrounding the slip bands, in exchange for the vacancies generated [37]. This creates a redistribution of matter and compression stresses within the slip band lamella, and tensile stresses within the metal matrix. This process of mean stress relaxation in the slip bands and the matrix is different, as the critical yield stress within the slip bands is lower than that of the metal matrix around the slip bands. With repetitive cycling, there is a cyclic creep within the lamella, and stress relaxation produced by the plastic strain formation of the extrusions. With the continuing exchange of mass between the slip bands and the matrix, the matrix eventually accumulates enough vacancies for the internal stress to reach the yield point, and stress relaxation takes place by intrusion formation [31]. Interestingly, damage to NiTi alloys also happens in a similar fashion by the formation of cracks on the surface due to fatigue in torsional loading [38].

## 4. Properties of Nickel–Titanium

**Figure 2:**
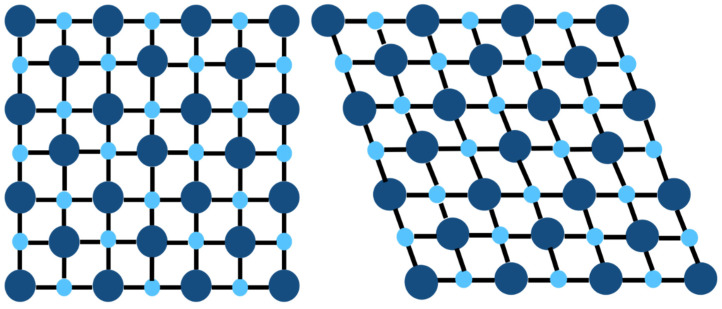
Austenitic (**left**) and martensitic (**right**) crystalline structures.

NiTi alloy is an equiatomic intermetallic compound, and its property of shape memory was first described in 1963 by Buehler. [39] At high temperatures, its crystals are in a simple cubic ‘austenite’ structure, and when cooled to below its transformational temperature, the austenite transforms to monoclinic ‘martensite’ crystals (Figure 2). This ability to change the crystalline phase reduces the stress at which it develops cracks, and makes NiTi more resistant to fatigue under local stresses [40]. Crack propagation in NiTi is related to martensitic transformation, rather than the presence of microscopic plastic deformation around the growing crack, which is found in other metallic alloys [41,42].

**Figure 3:**
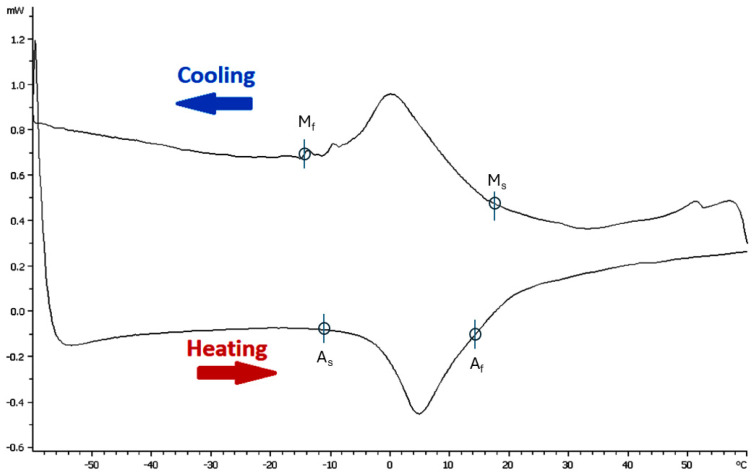
A DSC curve of untreated NiTi. Adapted with permission from Chien et al., 2023, under the Creative Commons Attribution (CC BY) license [43].

Stress and temperature both affect the stability of martensite, leading to both factors being able to induce its transformation. Differential scanning calorimetry (DSC) is used to determine the transformational temperatures for NiTi, and this is defined by four characteristic temperatures [43]. These temperatures are the martensitic start and finishing temperatures (M_s_, M_f_), as well as the respective austenitic start and finishing temperatures (A_s_, A_f_). Another descriptor is M_d_, which is the temperature above which martensite cannot be stress-induced from austenite. A sample DSC curve can be seen in Figure 3, and it denotes these characteristic temperatures.

**Figure 4:**
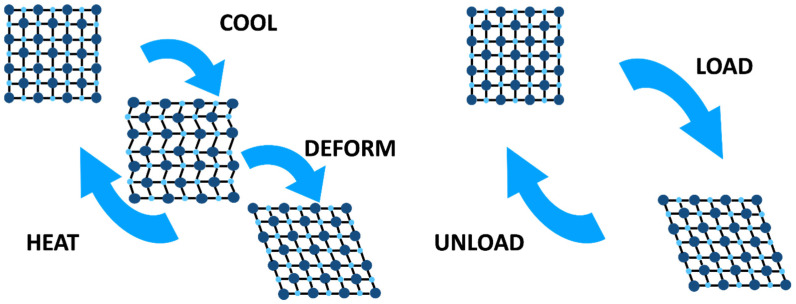
Schematics of thermal shape memory and superelasticity.

The transformation is a result of a slight shift in atomic position that changes the inter-atomic distance, leading to no sides of the crystal being the same length, and one angle being greater than 90° [44]. Martensite forms in multiple orientations with the same structure, and these are called *variants*; typically, two to four variants are formed side-by-side during transformation to accommodate the elastic strain around martensite, and this is known as *self-accommodation* [45]. When an austenitic NiTi alloy is cooled (see Figure 4), it forms martensitic twin variants. Because martensitic and austenitic crystals are of different crystalline shape and dimensions, martensite can only nucleate within the constraints of the austenitic phase by twinning. By twinning and forming martensite variants, the crystalline shape change can be accommodated, allowing for the formation of martensitic crystals without the accumulation of excessive strain energies [46]. The martensite structure formed has twin boundaries, which are free to move and are very low-energy. Under stress-induced martensite formation, transitional twinning happens at the martensite–austenite interface. This pattern of self-accommodation and transformation provides two unique properties to NiTi, namely *thermal shape memory and superelasticity* (also known as *pseudoelasticity or mechanical shape memory*) [47]. Of note, martensitic crystals formed during cooling or in response to stress are similar in shape. [46]

During its functioning, NiTi is typically loaded cyclically, and therefore fatigue is the most common mechanism of failure [48]. It has been shown that the structural fatigue in NiTi shape memory alloys does not differ from other metallic alloys in terms of crack initiation, propagation, and formation of intrusions and extrusions on the surface [49,50,51]. It is further understood that cracks initiate when NiTi undergoes phase transformation in the *active zone*, where there is localized phase transformation [52]. The reversible phase boundary motion between stress-induced martensite and austenite leads to permanent dislocation slip formation within the crystalline grain. This also occurs at the transformational interface between austenite and martensite, and between different martensite variants with different crystallographic orientations [53,54,55]. Taken together, dislocation is the consequence of unmatched deformation between austenite and martensite at the interface, which creates local stress fields that help with dislocation slip formation at this crystalline interface. Ultimately, this is the cause of what is known as *transformation-induced plasticity* (TRIP), and results in crack initiation in NiTi [56,57].

With cyclic strains, it was previously thought that there is an accumulation of dislocations in superelastic austenitic NiTi, which stabilizes the martensitic phase and means that it does not retransform back, or that the dislocations remain in the austenitic phase and make the transformation to martensite easier in the next cycle [38]. However, current models consider that due to accumulation of dislocation slips at transformational interfaces, there is locking-in of martensite within the stress fields that is created internally by dislocation slips [58,59]. This results in cyclic accumulation of residual strain within the material, which is called *transformational ratchetting* [60].

## 5. Rotary File Fracture

### 5.1. Factors Affecting File Fracture

Reasons for file fracture are multifactorial: its causes may be operator-, tooth-, or instrument-related. Clinical studies have reported varying incidences of unwinding and fracture of NiTi files of up to 38%, increasing with repeated use [11,61,62,63,64]. Collectively, studies suggest that the rate of defects/fractures of an instrument is affected by its size, taper, cross sectional shape, lack of operator proficiency, high torque motors, and length of pecking motion [11,65,66,67]. Studies have also consistently shown that an increased speed of rotation reduces the time it takes to fracture, because a file reaches its critical number of rotations to fracture more quickly [67,68,69]. Furthermore, instrument fracture is more likely in teeth with complex anatomy and in curved canals with instruments of greater taper [10].

**Figure 5:**
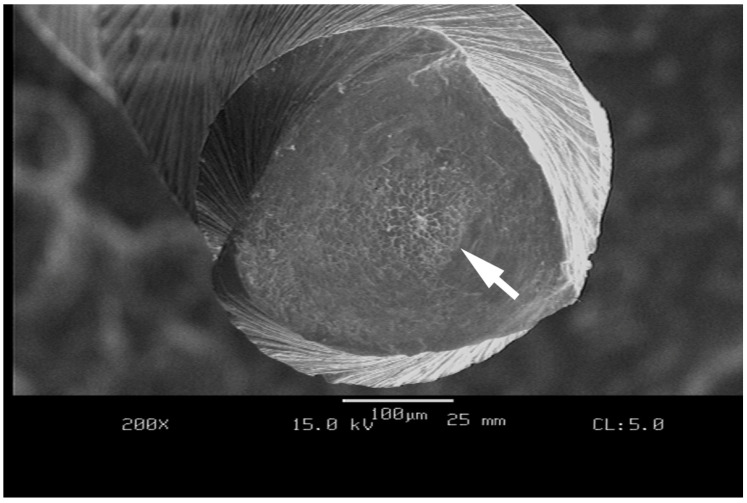
Scanning electron micrograph (SEM) of an as-received austenitic instrument fractured by torsional loading under ISO 3630-1 conditions. Note the area in the center of the instrument cross section with fracture-related dimples denoted by the white arrow.

Sattapan noted that more than 56% of Quantec 2000 rotary files (Kerr Sybron, Brea, CA, USA) fractured with unwinding or reverse winding of the spiral, characteristic of torsional fracture, and only a few fractured without any microscopic signs, due to flexural fatigue [70]. Characteristic dimple patterns are seen under a Scanning Electron Microscope (SEM) with files that fracture due to torsional resistance failure (Figure 5). It has been suggested that these appear from the nucleation of small voids, due to inclusions in the alloy which predispose the file to fracture [71,72].

So-called secondary phase particles and surface flaws on unused files and metal rollover at the cutting edges for used rotary NiTi instruments, and the dentine chips that lodge in these flaws, could contribute to crack propagation [73]. However, current evidence suggests that commercial medical-grade NiTi alloy is free of inclusions larger than 15–50 micrometers (µm), which is the critical flaw size for crack initiation, and hence they do not play any major role in fracture mechanism [74]. Moreover, typical machining marks on the surface of files after electropolishing have also been shown to be smaller than 3 µm [75]. This is again shorter than the critical crack length below which a material will not preferentially propagate a fatigue crack from the flaw.

One of the main reasons for the fracture of metals is crack initiation from persistent slip bands over its service life [76]. These slip bands form as a result of cyclic strain, which causes accumulation of dislocation slips at crystalline grain boundaries [23]. Fatigue as a result of cyclic straining has been shown to be an important reason for the fracture of NiTi files [77]. Fatigue failure is typically linked with no macroscopic warning signs of damage, and the fracture of NiTi files has been associated with no any signs of warning [78]. This would suggest that fracture of NiTi files is more often a result of overservice, rather than due to manufacturing defects.

### 5.2. Mechanisms of Endodontic File Fracture

#### 5.2.1. Flexural Fatigue

Common mechanisms of file fracture are torsional/shear failure, cyclic fatigue, or flexural fatigue. Pruett reported that a decrease in the root canal radius of curvature, or an increase in the angle of curvature, both reduced the number of cycles for which NiTi files could rotate prior to fracture, whereas revolutions per minute did not affect the flexural fatigue property of the files [79].

Flexural fatigue occurs when there are repeated cycles of tension and compression in a bent and rotating file, which causes structural breakdown and eventual fracture. Fatigue resistance is commonly tested in vitro in a curved metal tube, in a grooved block and rod assembly, in rotation against an inclined plane, or in a three-point bend fixture of a rotating instrument [80]. However, in vitro testing is limited by its post mortem analytical approach. According to a recent review, cyclic fatigue can be explored in silico via finite element analysis [81], but the same group of authors concluded that there were frank limitations to the utilization of finite element analysis in their own experiments [43]. Specifically, finite element analysis was unable to model crack initiation and propagation, and assumed a model of linear elastic deformation which does not account for the intermediary phases of NiTi. Consequently, it has been that suggested the use of the extended finite element method could be a more appropriate solution for in silico testing where theoretical models for ductile materials and crack propagation exist [82].

Most root canals are curved. Hence, there is an evident benefit of testing the flexural fatigue of rotary files; indeed, there have been numerous studies published in the last two decades on this mechanical property. To minimize the risk of the sudden fracture of a file due to flexural fatigue, it has been suggested to minimize the reuse of files, or to make the number of reuses dependent on the tooth being treated and curvature of the canal [73,83,84]. However, duration of use, visual observation, and number of uses do not correlate with the deterioration of NiTi instruments, which in fact is directly related to the forces and torque exerted on the instrument during its manipulation [85].

Flexural fatigue also detrimentally affects other properties of the file, like the torsional resistance [86,87,88,89,90,91,92]. It has been reported that 96.67% of deformed instruments collected after clinical use from specialist endodontic practice, with or without fracture, were affected by torsional failure, and this was the most common cause of fracture of rotary file. [93,94]

#### 5.2.2. Torsional Resistance

Oliet, in 1965, first described an equipment to test the torsional resistance of endodontic hand files. Torsional resistance is currently tested according to ANSI/ADA specification No.28 and ISO3630-1 [95]. In brief, the tip of the file is clamped at 3mm, and the file is rotated at 2rpm; then, the maximum torque and/or angular deflection of the file at fracture is recorded to assess the torsional resistance [95].

Torsional fracture occurs when the tip or any other part of an instrument binds to the canal wall, but the handpiece keeps turning. Twisting a file about its longitudinal axis at one end, whilst the other end is fixed, generates torsional stress. Torsional stress occurs in straight or curved canals, when resistance of the dentine against the canal wall results in friction and torsional force on file. Furthermore, once the elastic limit of the metal is exceeded, the rotary instrument undergoes plastic deformation (unwinding). The file will ultimately fracture if the load is sufficiently high; conversely, if the torsional strength of the file is increased, the incidence of breakage caused by torsional loading will decrease [12,96]. Torsional fracture in such a manner is of concern, because if torque is concentrated in one part of the instrument, it may be too high and induce a binding of the instrument, which is difficult to detect as resistance to rotation because it is barely perceptible to the practitioner [97].

The torsional resistance of a file depends on several factors. Hand files with a larger size and square cross section fracture at lower deflections than those with a smaller and triangular cross section. The torsional deflection is proportional to the modulus of rigidity and ductility, which varies with the material of the instrument, and also its work hardening during manufacturing [98]. It has been reported that hand files and reamers have a higher torsional resistance in a clockwise than in a counterclockwise rotation, as well as when there are fewer flutes per unit length [99,100,101,102,103]; the same is true for NiTi files [104]. The torque required at failure also increases with increased hand file size, although torque to fracture does not vary with the manufacturing process of either twisting files or machining files, but machined files are more stiff than twisted files [103].

In the 1980s, it was first shown that NiTi wires were less stiff in torsion compared to SS wires. NiTi files could undergo two and a half revolutions before fracturing, while SS files could only undergo one and three-fourths of a revolution [105,106]. In comparing hand SS and NiTi files, it was noted that SS files undergo a larger angular deflection prior to fracture, but the torque values at fracture are not significantly different for SS and NiTi files, and torque to fracture increases with an increased size of the file [104,107]. However, when comparing rotary conventional NiTi files, M-wire files, blue NiTi files of similar geometric design, and SS files, SS files reportedly have a higher torque to fracture, but there is no significant difference between the four in terms of their angle of deflection to fracture [108]. Conversely, SS files have a higher torque to failure than 0.2 taper NiTi files, but a lower torque to failure than 0.4 taper NiTi files [109].

For rotary NiTi files, the torque at fracture increases with increasing file size and reduced canal curvature [110,111,112,113]. It has also been reported that for rotary NiTi files, instruments with quadrilinear cross sections require higher torques to fracture in torsion compared to instruments with triangular cross sections [43]. A potential explanation for this finding is that as the inner core cross section of the file increases, it becomes more torque-resistant, requiring more torque to fracture, and undergoes less angular deflection at fracture [114,115,116]. Most newer-generation files made from heat-treated NiTi wire have a greater torsional resistance than conventional NiTi files [117,118].

It is evident from the available research that torsional resistance and torsional stresses are affected by the alloy properties [119], number of reuses [120], cross section [121,122], heat treatment [123,124], manufacturing mechanism [125], and pitch length of rotary files [126]. Torsional resistance is not the only cause of fracture by itself, but it could also reduce a rotating file’s cyclic fatigue resistance [87,88]. On the contrary, if the torsional pre-loading of files is below the superelastic limit, then it enhances the cyclic fatigue property of the file [127]. Moreover, suprathreshold cyclic preloading reduces the torsional resistance of conventional NiTi rotaries [128].

Torsional resistance also does not truly represent the durability of a file. The fracture of rotary files is more likely to be due to repetitive torsional stresses and flexural stresses [129]. Hence, a means to evaluate the build-up of internal mechanical “damage” within instruments after repeated use, and to predict the likelihood of failure, is of clinical significance [102].

#### 5.2.3. Torsional Fatigue

As discussed, fatigue failure is a result of a small flaw that forms during the fabrication, or, more likely, during the operation of the file, which then propagates as a crack due to repeated stress, a corrosive environment or both. This crack grows at a slower rate initially, and then propagates at a faster rate with time, until a sudden fracture happens when the crack reaches a critical size for the prevailing stress [20]. Regretfully, authors have erroneously used the label “torsional fatigue resistance” to describe resistance to torsional failure [130]. To be clear, ISO3630-1 tests refer to torsional failure as a sole entity; they do not have any cyclic component, but rather describe a single overloading event.

However, there is an entity that can be termed “torsional fatigue” as a distinct third fracture mechanism besides cyclic fatigue and torsional failure. This phenomenon was perhaps first discussed by Best in 2004, who reported that greater angular deflection of files led to greater tension-compression-shear forces, which in turn caused faster crack propagation and consequently file fracture at a lower number of rotations [131].

When the rotary file engages dentine, the part following the cutting edge undergoes compression, and the part of the file preceding the engaged cutting edge undergoes tension [132]. As the cutting area is blocked by dentine, the coronal part of the instrument, which is moved by the handpiece, keeps rotating. Due to the superelasticity of NiTi, the file undergoes some torsion, and as the developed torque increases, the instrument cuts part of the dentine, eliminating the blockage. With the release of the instrument, torsion disappears again due to the elasticity of the instrument [133]. But, with fatigue, instruments may fail with load amplitudes lower than the yield strength [134]. Additionally, the load generated on the instrument is affected not by the shape of the canal, but by the working area of the instrument, and during continued rotation, the instrument works under stress from the beginning of rotation, due to the rotation speed [135].

To test torsional fatigue, the set-up is somewhat like that of torsional resistance, but in the case of torsional fatigue (repetitive torsional resistance or dynamic torsional resistance), the file is allowed to rotate to a certain set amount of angular deflection or set torque levels, then is reversed to zero angular deflection or zero torque levels, and the process is repeated until fracture, to measure the torsional fatigue resistance [129,131,136]. The limited number of available studies on torsional fatigue suggests that similar cross section files can have different torsional fatigue. Flex Master has been shown to have better torsional fatigue resistance than ProTaper, and convex-triangular cross section files to have better resistance than equilateral triangular cross section and R-phase twisted files. To investigate this, researchers took a measurement at D5 (5mm from tip of the file), with light-cured resin used to clamp the tip of the file, utilizing 1Ncm pre-set torque in the motor rotating at 300rpm, and the test allowed the files to rotate until they reached pre-set torque [136,137].

Abu-Tahun et al. noted that V-taper files made from conventional NiTi had a significantly higher number of cycles to failure at a pre-set torque, followed by heat-treated V-Taper2H, than HyFlex EDM and HyFlex CM [138]. Similarly, it was reported that rotary NiTi files with an increased inner-core diameter can undergo a higher number of cycles at a pre-set torque and speed prior to fracture than thinner files can, making the FlexMaster file more torsional-fatigue-resistant than the XP-Endo Shaper, TruShape and ProFile Vortex [139]. With same methodology, FlexMaster was again reported to have the highest number of cycles to failure, followed by Vortex Blue, TruNatomy, and then HyFlex CM [140]. Similarly, torsional fatigue also reduces the resistance of files to cyclic fatigue [141].

The torsional failure of rotary instruments should be investigated not only for the maximum torsional strength and the angular distortion at break, but also for the yield strength and toughness that contribute to the resistance of the instrument to torsional breakage [129]. Yum tested the torsional toughness of files and noted that most files plastically deformed prior to fracturing due to torsion. However, their yield strength and fracture toughness varied, and they both contributed to the resistance of the instrument to torsional fracture [96,129].

While torsional resistance is an important property for hand files, torsional fatigue becomes more relevant for continuous-rotation NiTi files for the assessment of safety, especially when the risk of taper lock is minimal, as with the newer endodontic motors with auto-stop or auto-reverse torque-control function. In fact, with auto-reverse, there is a higher risk of repetitive torsional loading, as well as an increased risk of failure due to torsional fatigue [80,129,138].

Newer instrument and wire manufacturing technology has not only permitted new instrument designs; it has also improved the overall physical characteristics of endodontic files [2]. It has been further suggested that greater operator experience, extensive preclinical training and torque-controlled motors are related to less taper lock [65,142]. But this does not affect the torsional fatigue failure of the rotary file, and there is a scarcity of reports on the torsional fatigue of NiTi rotary files. Many earlier studies have investigated torsional resistance, but torsional fatigue may be more important clinically for the following reasons:

- Torque-control motors prevent files from reaching the ultimate torsional strength to fracture.
- A helix angle in files and a non-cutting tip prevent threading-in of the files, reducing the likelihood of it reaching ultimate strength.
- Rotary files are used with vertical amplitudes with in-and-out or brushing motions, further reducing the risk of file binding, as it is not forced and follows a glide path created by the hand file.
- In a clinical scenario, files rotate much faster than at 2rpm, the rotational speed specified by the ISO norm.

Consequently, future work should consider torsional fatigue as a relevant variable. Test fixtures, and potentially norms, should be developed that allow manufacturers and clinicians to understand the limitations and possible specific usage patterns related to torsional fatigue.

## 6. Conclusions

Root canal preparation with NiTi files is easier to perform, and results in better maintenance of the original canal shape. The uptake of rotary NiTi files has increased over the last two decades, with evidence suggesting fewer procedural errors by inexperienced operators with the use of contemporary instruments, and an increased success rate over a short-term period. Therefore, it is important to understand different fire fracture mechanisms to prevent adverse events and promote confidence for the user. Flexible files are vulnerable to torsional stress, but more resistant to cyclic fatigue. However, torsional fatigue remains the least researched and understood aspect of NiTi file fracture, and calls for further research to complete our understanding of fracture mechanics.

## Figures and Tables

**Table 1:** List of definitions.

Elastic modulus	The stiffness or resistance to deformation of a material, which is calculated as the ratio of elastic stress to elastic strain [18].
Flexibility	The reciprocal property to stiffness of the material, which is the deformation obtained per unit of applied stress [19]. Flexibility may also be referred to as *springiness.*
Elastic limit	The stress at which some permanent deformation occurs (deformation is not recovered on unloading) [19].
Plastic deformation	Occurs when a load applied to a material takes it past the elastic limit, and the material does not return to zero strain on removing the load. This phenomenon occurs when some atoms or molecules cannot return to their original position on removing the load because they have gone past an energy maximum, and they continue to remain in their new position to become stable at that position [19].
Brittleness	The relative inability of a material to deform plastically before it fractures [18].
Ductility	The amount of plastic strain produced in the specimen at fracture due to tensile stress is called the *ductility* of the material [19].It is reported as percentage elongation.
Yield strength and ultimate strength	Yield strength is the maximum stress that a structure can withstand without sustaining a specific amount of plastic strain, and ultimate strength is the stress at the point of fracture [18].
Resilience	The ability of a metal to absorb energy when elastically deformed and then return it when it is unloaded. It is measured by the modulus of resilience, which is the strain energy per unit of volume required to stress the material from zero to yield stress [20].
Toughness	The ability of a material to absorb energy in the plastic range, measured as the total area under the stress–strain curve; hence, toughness is a function of both ductility and strength [20].
Fracture toughness	The ability of a material to absorb and/or dissipate energy, due to the applied stress, by elastic and plastic deformation before fracturing. This is measured by introducing a crack of known size and shape, and then measuring the stress required for this crack to grow [19].
Shear	Shear stress tends to resist the sliding or twisting of one portion of a body over another, where the layers of atoms or molecules of the material are envisaged as sliding over one another [19]. Shear stress can also be produced by a twisting or torsional action on a material [18].
Torsion	Rotational motion about the longitudinal axis of one end of the member relative to the other end. In torsion, each element of the material deforms in pure shear. The shear strain is directly related to the radius of the file to which it is clamped and the angle from the shank to the point at which it is measured, and inversely related to length of the file from the shank to the point of clamping [20].
Fatigue	The process of progressive, localized, and permanent structural change occurring in a material subjected to conditions that produce fluctuating stresses and strains at some point (or points), and that may culminate in cracks or complete fracture after a sufficient number of fluctuations [20].
Fatigue limit	Most materials show a continuously declining stress for failure as the number of cycles increases, but some materials show a stress below which no amount of load cycling produces a failure; this is the fatigue limit, or endurance limit [19].

## Data Availability

The original contributions presented in this study are included in the article. Further inquires can be directed to the corresponding authors.
